# Type I interferon signaling is required for the APOBEC3/Rfv3-dependent neutralizing antibody response but not innate retrovirus restriction

**DOI:** 10.1186/s12977-017-0349-2

**Published:** 2017-04-17

**Authors:** Bradley S. Barrett, Michael S. Harper, Sean T. Jones, Kejun Guo, Karl J. Heilman, Ross M. Kedl, Kim J. Hasenkrug, Mario L. Santiago

**Affiliations:** 1 Department of Medicine, University of Colorado Denver, Aurora, CO USA; 2 Department of Immunology and Microbiology, University of Colorado Denver, Aurora, CO USA; 3 Rocky Mountain Laboratories, NIAID, NIH, Hamilton, MT USA; 4 Division of Infectious Diseases, University of Colorado Denver, Mail Stop B-168, 12700 E 19th Avenue, Aurora, CO 80045 USA

**Keywords:** IFNAR, Friend retrovirus, Neutralizing antibody, Deaminase, LDV

## Abstract

**Background:**

APOBEC3/Rfv3 restricts acute Friend retrovirus (FV) infection and promotes virus-specific neutralizing antibody (NAb) responses. Classical Rfv3 studies utilized FV stocks containing lactate-dehydrogenase elevating virus (LDV), a potent type I interferon inducer. Previously, we showed that APOBEC3 is required for the anti-FV activity of exogenous IFN-alpha treatment. Thus, type I interferon receptor (IFNAR) signaling may be required for the APOBEC3/Rfv3 response.

**Results:**

To test if the APOBEC3/Rfv3 response is dependent on type I IFN signaling, we infected IFNAR knockout versus IFNAR/APOBEC3 double-knockout mice with FV/LDV or LDV-free FV, and evaluated acute FV infection and subsequent NAb titers. We show that LDV co-infection and type I IFN signaling are not required for innate APOBEC3-mediated restriction. By contrast, removal of LDV and/or type I IFN signaling abrogated the APOBEC3-dependent NAb response.

**Conclusions:**

APOBEC3 can restrict retroviruses in a type I IFN-independent manner in vivo. By contrast, the ability of APOBEC3 to promote NAb responses is type I IFN-dependent. These findings reveal novel insights on the interplay between type I IFNs and APOBEC3 in vivo that may have implications for augmenting antiretroviral NAb responses.

**Electronic supplementary material:**

The online version of this article (doi:10.1186/s12977-017-0349-2) contains supplementary material, which is available to authorized users.

## Background

Innate immune mechanisms provide a means for the host to control pathogens before more slowly developing adaptive immune responses come into play. Innate immunity was linked to the production of type I interferons (IFN), which orchestrate an antiviral state through the expression of hundreds of interferon-stimulated genes (ISGs) [1]. Some ISGs encoded proteins known as ‘restriction factors’, which directly inhibit invading pathogens. These restriction factors include the seven human APOBEC3 enzymes (hA3A to hA3H): deoxycytidine deaminases that counteract a broad range of retroviruses including HIV-1 [2, 3], and exhibit a wide range of sensitivities to type I IFN induction [4–7]. In the absence of the HIV-encoded antagonist Vif, APOBEC3 gets incorporated into budding HIV-1 particles, inhibiting replication in the next target cell either by physically impeding reverse transcription or hypermutating single-stranded reverse transcripts [8]. However, the majority of APOBEC3 studies involved the ectopic expression of APOBEC3 in cell lines, which may not be physiologically relevant. In particular, transfecting APOBEC3 expression constructs into cells would bypass potential upstream regulatory pathways such as type I IFN signaling that may be required for APOBEC3 to restrict retroviral replication in vivo.

In contrast to humans, mice encode only one APOBEC3 gene, mA3. Thus, mA3 knockout (KO) mice provided the field with a powerful means to understand the in vivo impact of APOBEC3 on retroviral pathogenesis and immunity [9–12]. Our group utilized the murine Friend retrovirus (FV) infection model, which was key to the identification of host genes that control retrovirus infection, including the first retrovirus restriction factor, Fv1 [13, 14]. FV is a complex of a replication-competent Friend murine leukemia virus (F-MuLV) and a replication-defective spleen-focus forming virus (SFFV) that causes splenomegaly and erythroleukemia [15]. We previously showed that C57BL/6 (B6) wild-type (WT) and mA3 KO mice had similar levels of plasma viral load at 7 days post-infection (dpi), but the infectivity of the virions was significantly higher in mA3 KO mice [10, 16–18]. Importantly, administration of recombinant IFNα inhibited FV replication in WT mice, but exogenously administered IFNα had no antiviral effect in mA3 KO mice [17]. These findings indicated that mA3 acts downstream of exogenous IFNα to inhibit FV replication. However, it remains unclear if mA3 can inhibit retroviral infection in vivo in the absence of endogenous type I IFN signaling.

The impact of mA3 extends beyond retrovirus restriction. Our group and others reported that mA3 encoded Rfv3, a classical resistance gene in B6 mice that promoted recovery from FV viremia by stimulating a stronger NAb response [10, 11, 19, 20]. These early studies on Rfv3 were conducted in F_1_ hybrid mice harboring the *Fv2* susceptibility allele from BALB or A.BY strains, which promotes higher FV replication by driving erythroblast proliferation [21–23]. Nevertheless, the impact of mA3/Rfv3 on NAb responses was also observed in pure B6 mice. Compared to B6 WT mice, B6 mA3 KO mice had significantly lower FV-specific NAb responses by 28 days post-infection (dpi) [10]. The underlying mechanism was multifaceted. Compared to mA3 KO mice, WT mice exhibited: (1) enhanced germinal center (GC) responses due to noninfectious virion particle release [16]; (2) augmented GC responses due to contraction of the marginal zone B cell compartment [24]; (3) higher levels of antiviral IgG2b and IgG2c antibodies [25]; and (4) enhanced somatic hypermutation of virus-specific IgG antibodies [26]. Antibody neutralization was dependent on Fcγ receptors, as removal of the common γ chain (FcRγ) and particularly FcγRIV, which bind to IgG2b and IgG2c antibodies, rendered 28 dpi antisera incapable of neutralizing FV in vivo [25]. By contrast, removal of complement C3 had no effect on the in vivo neutralization capacity of 28 dpi antisera from mA3-sufficient mice [25].

Interestingly, Rfv3 was initially discovered using FV stocks that contained lactate-dehydrogenase elevating virus (LDV), an endemic RNA virus in wild mouse populations and component of the murine ‘virome’ [27, 28]. Since Rfv3 was discovered using FV/LDV stocks, our studies on the role of mA3 in NAb responses utilized FV/LDV [10, 16, 20, 26]. LDV has potent immunostimulatory properties; thus, data obtained using FV/LDV may not necessarily be reproduced using ‘LDV-free’ FV. LDV can suppress T and B cell responses in FV infection [29–31], and can induce high levels of type I IFNs through Toll-like receptor 7 (TLR7) sensing [32]. LDV-free FV infection of B6 mice resulted in very low or undetectable IFNα expression compared to FV/LDV co-infection [33]. Notably, type I IFNs can also augment and shape humoral immune responses in vivo [34–37]. In certain contexts, LDV may also enhance antibody responses [38, 39]. Thus, we hypothesized that type I IFN signaling might be required for the mA3/Rfv3-dependent NAb response during FV/LDV infection.

In order to investigate the impact of type I IFN signaling in mA3 restriction and NAb responses, we prepared mice doubly-deficient in mA3 and the type I IFN receptor (IFNAR). IFNAR is a heterodimer consisting of IFNAR-1 and IFNAR-2 subunits that together form a binding site for the antiviral cytokines IFNβ and IFNα subtypes [40]. IFNAR KO mice lacked the IFNAR-1 receptor chain and were unresponsive to JAK/STAT signaling cascades triggered by type I IFNs [41]. Many viral infections, including FV, replicated to significantly higher levels in IFNAR KO compared to WT mice [41, 42]. However, the downstream effector mechanisms remain under intense investigation. In this report, we tested whether mA3 can inhibit FV and FV/LDV infection and promote NAb responses in the absence of IFNAR signaling.

## Results

### Murine APOBEC3 inhibited infectious virus release in the absence of type I IFN signaling

**Fig. 1:**
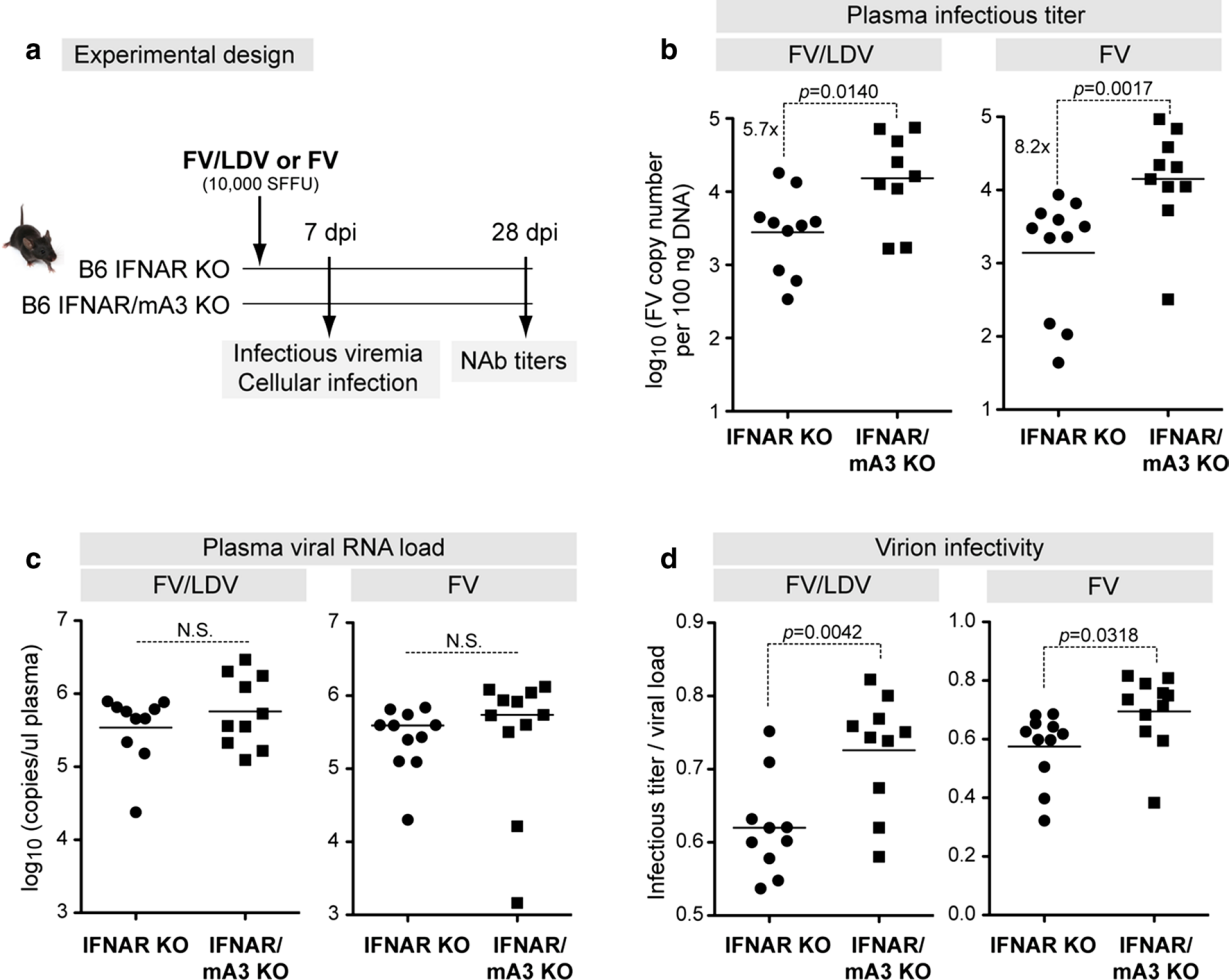
APOBEC3/Rfv3 inhibits infectious virus release in an IFNAR KO background. **a** Experimental design. B6 IFNAR KO and IFNAR/mA3 dKO mice were infected with FV and samples analyzed at the indicated time points. **b** Infectious viremia was measured by incubating plasma onto susceptible *Mus dunni* cells for 2 days and determining F-MuLV proviral DNA levels. Infectious viremia was determined for both (*left*) FV/LDV and (*right*) LDV-free FV infection. The same samples in (**b**) were used to determine **c** plasma viral RNA loads by qPCR. The ratio of the log-transformed infectious titer in (**b**) and plasma viral loads in (**c**) were used to estimate **d** virion infectivity. Each *dot* corresponds to a mouse and *lines* correspond to mean values. The total number of mice analyzed was combined from 2 to 3 independent experiments. Data were analyzed using a 2-tailed unpaired Student’s *t* test, with exact *p* values shown. Fold-change values in statistically-significant comparisons were based on average non-log-transformed values

We previously demonstrated that B6 mA3 KO mice had higher infectious viremia compared to wild-type (WT) mice in experimental infections using LDV-containing FV stocks (FV/LDV) [10, 16] or LDV-free FV stocks (FV) [18, 43]. These data suggested that the induction of type I IFNs by LDV might not be required for mA3 to inhibit FV infection in vivo. To test this hypothesis directly, IFNAR KO and IFNAR/mA3 double KO (dKO) mice (<1 year old) were infected i.v. with 10^4^ spleen focus forming units (SFFU) of FV/LDV or FV (Fig. 1a). At 7 days post-infection (dpi), infectious viremia in the plasma was evaluated. Plasma infectious viremia was 5 to eightfold higher in IFNAR/mA3 dKO mice compared to IFNAR KO mice in both FV/LDV (Fig. 1b, left panel) and FV (Fig. 1b, right panel) infections. We next quantified the levels of viral RNA in the plasma (Fig. 1c), an indirect measure of the total number of particles released. Consistent with our previous findings on WT versus mA3 KO mice [17], we found no significant difference in plasma viral RNA load between IFNAR and IFNAR/mA3 dKO mice at 7 dpi in both FV/LDV and FV infections (Fig. 1c). The ratio of infectious titers (Fig. 1b) and plasma viral RNA load (Fig. 1c) provides a measure of virion infectivity [16, 17]. As shown in Fig. 1d, virions in the 7 dpi plasma of IFNAR/mA3 dKO mice had significantly higher infectivity than those from IFNAR KO mice. Our findings reveal that type I IFN signaling and LDV co-infection are not required for mA3-mediated inhibition of virion infectivity during acute infection.

### IFNAR signaling is not required for mA3 restriction of acute FV/LDV or FV infection

**Fig. 2:**
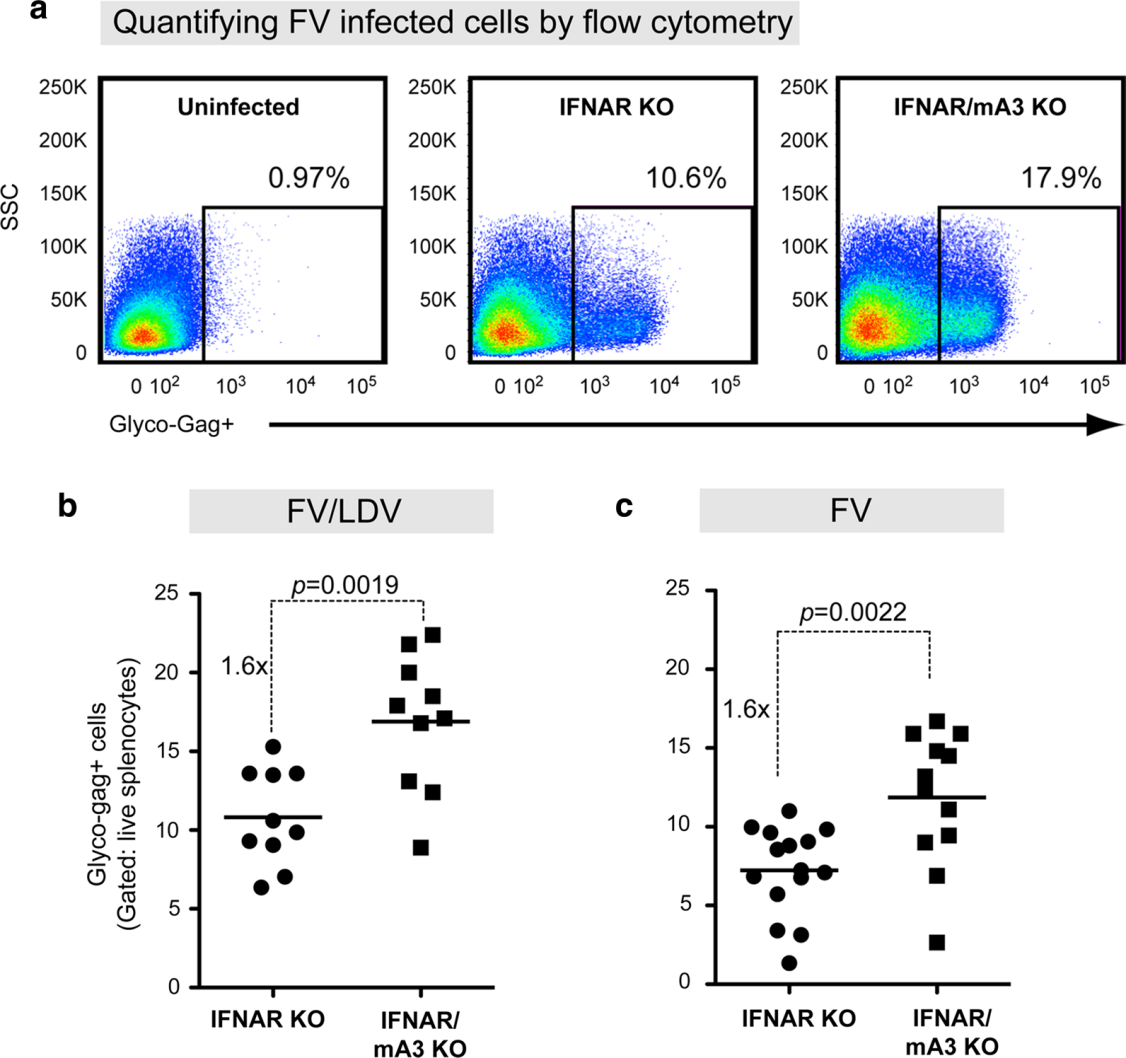
APOBEC3/Rfv3 inhibits acute FV infection of splenocytes independent of type I IFN signaling. Splenocyte FV infection levels were measured by flow cytometry using a glyco-gag specific monoclonal antibody. **a** Representative flow plots showing glyco-gag+ splenocytes from FV/LDV infected mice at 7 dpi. The percentage of live splenocytes that expressed glyco-gag was evaluated in **b** FV/LDV and **c** LDV-free FV infections. Each *dot* corresponds to a mouse and *lines* correspond to mean values. The total number of mice analyzed was combined from 2 to 3 independent experiments. Fold-change of mean values per cohort are shown. Data were analyzed using a 2-tailed unpaired Student’s *t* test, with exact *p* values shown

To complement the plasma viremia results, we evaluated cellular F-MuLV infection levels in splenocytes. To detect F-MuLV infected cells, we utilized a previously described flow cytometry method using MAb 34, which is specific for the F-MuLV glyco-gag protein [16, 44] (Fig. 2a). Flow cytometry-based methods to detect virus infection generally have lower dynamic range than quantitative PCR-based methods. Nevertheless, consistent with the plasma infectious viremia data, we observed that in both FV/LDV (Fig. 2b) and FV (Fig. 2c) infections, mA3 deficiency in IFNAR KO mice resulted in significantly higher splenocyte infection levels (1.6-fold) compared to IFNAR KO mice. Altogether, the data in Figs. 1 and 2 indicate that mA3 restricted acute FV replication independently of type I IFN signaling and LDV co-infection.

### LDV co-infection is required for the mA3/Rfv3 neutralizing antibody phenotype

**Fig. 3:**
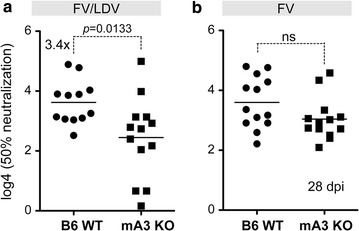
LDV co-infection is critical for the APOBEC3/Rfv3-dependent NAb response. B6 WT and mA3 KO mice were infected with 10^4^ SFFU of **a** FV/LDV or **b** LDV-free FV. Plasma samples at 28 dpi were heat-inactivated and the reciprocal plasma dilution that conferred 50% neutralization was computed. Log_4_-transformed data are shown and used for statistical analyses. Each *dot* corresponds to a mouse and *lines* correspond to mean values. The total number of mice analyzed was combined from 2 independent experiments. Fold-change values in statistically-significant comparisons were based on median non-log-transformed values. Data were analyzed using 2-tailed unpaired Student’s *t* test, with *p* values indicated; ns, not significant (*p* > 0.05)

The Rfv3 gene was identified using FV/LDV stocks [21]. LDV is a potent stimulator of type I IFN responses [32], and type I IFNs can stimulate B cell responses [34–37, 45]. To test if mA3/Rfv3-mediated enhancement of NAb responses occurs in LDV-free FV infection, WT and mA3 KO mice were infected with 10^4^ SFFU of FV/LDV or FV and plasma NAb levels were evaluated at 28 dpi. Consistent with our previously published data [10, 25], mA3 KO mice had significantly lower NAb titers (3.4-fold) compared to WT mice during FV/LDV co-infection (Fig. 3a). By contrast, the difference in NAb responses between WT and mA3 KO mice infected with LDV-free FV was not statistically significant (Fig. 3b). Thus, LDV co-infection is required for mA3 to promote the FV-specific NAb response.

### IFNAR signaling is required for the mA3/Rfv3-dependent neutralizing antibody response

**Fig. 4:**
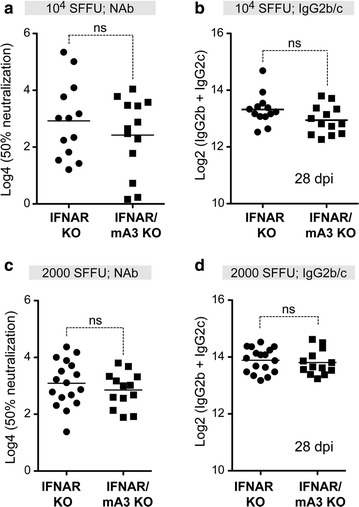
APOBEC3/Rfv3-dependent NAb response requires type I IFN signaling. Mice were infected with FV/LDV at two different inoculum doses: **a**, **b** 10,000 SFFU and **c**, **d** 2000 SFFU. **a**, **c** Plasma samples at 28 dpi were heat-inactivated and the reciprocal plasma dilution that conferred 50% neutralization was computed. Log_4_-transformed data are shown and used for statistical analyses. **b**, **d** FV-specific IgG2b/c titers were determined by endpoint-titration ELISA for mice infected with 10^4^ SFFU of FV/LDV. Native FV virions were coated into ELISA plates and twofold dilutions of plasma were added. IgG2b/c antibodies were detected using a combination of anti-IgG2b and anti-IgG2c conjugates. In all panels, each *dot* corresponds to a mouse and *lines* correspond to mean values. The total number of mice analyzed was combined from 2 independent experiments. Data were analyzed using 2-tailed unpaired Student’s *t* test; *ns* not significant (*p* > 0.05)

Since type I IFNs can influence antibody responses and LDV is a potent inducer of type I IFNs, we next investigated if type I IFN signaling is required for the mA3/Rfv3-dependent NAb response. IFNAR KO and IFNAR/mA3 KO mice were infected with 10^4^ SFFU of FV/LDV and NAb titers evaluated from 28 dpi plasma. In contrast to FV/LDV infections showing that mA3 influences NAb responses in the B6 background (Fig. 3a) [10, 25], the NAb titers between IFNAR KO and IFNAR/mA3 KO mice were not significantly different from each other (Fig. 4a).

We previously showed that the mA3-dependent NAb response correlated with higher titers of FV-specific IgG2b and IgG2c, both of which signal through FcγRIV [25, 46]. We therefore quantified FV-specific endpoint IgG2 titers at 28 dpi plasma of FV/LDV-infected IFNAR KO versus IFNAR/mA3 KO mice. In contrast to mA3 influencing antiretroviral IgG2b and IgG2c titers, mA3 deficiency had no impact on the FV-specific IgG2 response in an IFNAR KO background (Fig. 4b). These data suggest that type I IFN signaling modulates the APOBEC3/Rfv3-dependent, FV-specific IgG2b/c response.

IFNAR KO mice are more susceptible to FV infection compared to normal B6 mice [42]. IFNAR KO mice infected with 10^4^ SFFU of FV had viral loads that were 250-fold higher than B6 WT mice infected with the same inoculum dose. Thus, the observation that mA3 can influence NAb responses in the B6 background (Fig. 3a) but not in an IFNAR KO background (Fig. 4a) may be due to a difference in infection levels and/or antigen load. Specifically, higher FV titers may result in greater immunosuppression in the IFNAR KO background. To address this caveat, we infected IFNAR KO mice with fivefold lower FV inoculum dose (2000 SFFU) and evaluated NAb titers at 28 dpi. A 2000 SFFU inoculum resulted in FV viral loads in IFNAR KO mice that were comparable to that of B6 WT mice infected with 10^4^ SFFU at 7 dpi (≤10^3^ copies/ml of F-MuLV). At this lower inoculum dose, we did not observe a significant difference in NAb titers (Fig. 4c) and FV-specific IgG2b/c titers (Fig. 4d) between IFNAR KO and IFNAR/mA3 double-KO mice. Combining the results from Figs. 3 and 4c in a four-way comparison, removal of IFNAR signaling in mA3 KO mice did not lead to a further decrease in NAb responses (Additional file 1: Fig. S1). This data suggested that IFNAR and mA3 function in a linked pathway controlling NAb responses. Altogether, the data demonstrates that the mA3-dependent antibody response against FV/LDV infection requires type I IFN signaling in vivo.

## Discussion

Type I IFN signaling is required for controlling many viral infections, but the downstream mechanisms remain less understood. Recently, major efforts have been undertaken to determine which of the hundreds of ISGs act as antiviral effectors in vivo. Several APOBEC3 genes are considered as ISGs. Thus, we hypothesized that APOBEC3 may be a critical effector of endogenous type I IFN response against FV infection. Surprisingly, we found that mA3 acts as a type I IFN-independent restriction factor that limits acute FV infection. A likely explanation is that B6 mice already express high baseline expression levels of mA3 [47–49], potentially mitigating the need for type I IFN-mediated induction of mA3 to achieve restriction. However, the result is intriguing given that mA3 is critical for the inhibitory activity of recombinant IFNα treatment against FV infection in vivo [17]. Collectively, our findings suggest that the effector mechanisms mobilized by exogenous IFNα administration (primarily APOBEC3) might be different from that of an endogenous type I IFN response (APOBEC3 + other ISGs) during retrovirus infection. We speculate that the difference in ISG effector(s) mobilized during exogenous IFNα treatment versus the endogenous type I IFN response may be due to the nature of the type I IFNs involved. Our previous study involving exogenous IFNα treatment utilized only one type of IFNα (universal) [17], but an endogenous type I IFN response likely stimulates a combination of multiple IFNα subtypes. The IFNα subtypes demonstrate diverse biological properties, including antiviral activities, both in vitro and in vivo [50, 51]. To date, it remains unclear if diverse IFNα subtypes may be stimulating distinct antiviral effectors in vivo. The antiviral ISG effectors that act downstream of IFNAR signaling to reduce FV replication in vivo remains to be determined.

In contrast to mA3 functioning as a type I IFN-independent restriction factor that limits acute infection, mA3 functioned as a type I IFN-*dependent* innate resistance factor that promotes virus-specific NAb and IgG2 responses. The reason for why mA3 requires type I IFN signaling to modulate antibody responses but not retroviral restriction may be due to differences in the complexity of these processes. Retrovirus restriction requires only the APOBEC3 enzyme, whereas orchestrating a NAb response would require the mobilization of multiple pathways. For example, type I IFNs can modulate the contribution of follicular B cells to the antibody response, resulting in higher antigen-specific IgG2c titers [45].

LDV, a potent type I IFN inducer, was also required to reveal the Rfv3 phenotype. At first glance, this finding appears to be at odds with data showing that LDV can suppress B cell responses during FV infection [31]. FV/LDV infections exhibited a delay in NAb development compared to LDV-free FV infections in B6 mice [31]. However, this previous study utilized fivefold to tenfold lower levels of viral inoculum compared to our current study. In fact, early studies suggested that under some conditions, LDV may also enhance antibody responses [38, 39]. Thus, we speculate that at low inoculum doses, LDV may suppress adaptive immune responses. By contrast, at high inoculum doses (such as the one we used in this study), LDV’s immunosuppressive effects may have been overcome. Interestingly, FV on its own can suppress B cell responses through PTEN-mediated inhibition of the PI3K pathway [52]. Further studies should help shed insight on how FV and LDV inoculum dose affects the balance between B cell stimulation and suppression in the FV/LDV coinfection model. Overall, the IFNAR-dependence of the mA3/Rfv3 NAb response in the current study suggested that the type I IFN response induced by LDV also activated mA3. This raises the possibility that mimicking the ‘adjuvant’ properties of LDV through type I IFN-inducing agents may augment antiretroviral NAb responses by modulating APOBEC3 activity.

## Methods

### Mice

C57BL/6 (B6) and IFNAR KO mice [41], backcrossed for over 15 generations in the B6 background, were purchased from the Jackson Laboratory. mA3 KO mice, generously provided by Dr. Warner Greene, were initially derived from a 129P2 embryonic stem cell gene trap library [10] and backcrossed for 10 generations into B6. The IFNAR and mA3 genes are located on chromosomes 16 and 15, respectively. To generate IFNAR/mA3 dKO mice, IFNAR KO and mA3 KO mice were crossed and the progeny genotyped to generate IFNAR^+/−^ mA3^+/−^ mice. These heterozygous mice were further crossed to obtain IFNAR^−/−^ mA3^−/−^ mice (~6.25% by Mendelian genetics). The cohorts described in this study specifically compared IFNAR KO versus IFNAR/mA3 dKO mice less than 6 months of age.

### FV infections

Two FV stocks were used in this study. FV/LDV was in vivo passaged from the FV stock that was used to initially describe Rfv3 [21]. LDV-free FV (or simply FV) was in vitro passaged, and confirmed to have no contaminating LDV [30]. Both stocks were prepared in BALB/c mice and titered as previously described. FV (2000 to 10,000 SFFU) was inoculated intravenously through the retroorbital sinus in 300 µl RPMI and bleeds or terminal harvests were obtained at either 7 or 28 days post-infection.

### Plasma virus infectious titers

As previously described [17, 18], 5 µl of plasma were incubated with *Mus dunni* cells in a 48-well plate, and after 2 days, F-MuLV DNA copies were measured by quantitative PCR normalized to 100 ng total DNA input.

### Plasma viral load quantification

Viral RNA was extracted from 50 µl of plasma using the RNAEasy kit (Qiagen). The isolated RNA was then subjected to a one-step TaqMan reverse transcriptase PCR reaction using FV-specific primers as previously described [17, 18]. FV copy numbers were determined against a standard curve using T7-transcribed RNA standards.

### Flow cytometry

Splenocytes were stained for the F-MuLV glyco-gag protein using the mAb 34 antibody for 1 h, then stained with anti-mouse IgG1-APC (Columbia Biosciences) for 30 min. An LSR-II flow cytometer (BD Biosciences) was used to capture up to 250,000 events per sample, and Flowjo software (Treestar) was used to analyze the data. Glyco-gag+ cells were gated based on biological controls using uninfected splenocytes (Fig. 2a).

### Neutralizing antibody assay

Serial dilutions of heat-inactivated plasma were combined with a standard amount (50–100 infectious units) of F-MuLV for 1 h at 37 °C, then the mixture was added onto *Mus dunni* cells in 48-well plates. After 2 days, F-MuLV titers were detected using an F-MuLV Env-specific monoclonal antibody, mAb 720 [10, 53]. Inhibition curves were constructed by nonlinear regression using the one-site total equation in GraphPad Prism 5.0 [16]. NAb titers corresponded to the concentration of plasma that resulted in 50% neutralization compared to the control samples with F-MuLV alone.

### FV-specific IgG2b/c titers

Endpoint titration ELISAs were performed as previously described [16, 25]. Briefly, serial twofold dilutions of plasma were incubated for 1 h on 96-well Immulon-4 HBX plates pre-coated with 200 ng native FV virions. After 6 washes with PBS-Tween 10, an equimolar combination of anti-IgG2b and anti-IgG2c antibodies (1:4000; Southern Biotechnology) conjugated to HRP were added, washed, then incubated with TMB substrate. Endpoint titers were calculated as the plasma dilution that corresponded to twice the mean background of wells without plasma added.

### Statistical analyses

Infection and antibody values were log-transformed to normalize the data for analysis using a 2-tailed unpaired Student’s *t* test (GraphPad Prism 5.0).

## Additional file

**Additional file 1: Fig. S1.** Four-way comparison of NAb responses. NAb data from WT versus mA3 KO mice from Fig. 3 and IFNAR KO versus IFNAR/mA3 dKO mice from Fig. 4c were analyzed. Inoculum doses were lower in the IFNAR KO background to account for the higher susceptibility of these mouse strains to FV infection. Pairwise analyses were performed using a 2-tailed Student’s *t* test. *p < 0.05; **p < 0.01; *NS* not significant.
