# Supplementary material for: Type I interferon signaling is required for the APOBEC3/Rfv3-dependent neutralizing antibody response but not innate retrovirus restriction
Source: Retrovirology. 2017 Apr 17;14:25. doi: 10.1186/s12977-017-0349-2 (PMC5392950; doi:10.1186/s12977-017-0349-2)
Supplement: Supplementary file 1 — Additional file 1: Fig. S1. Four-way comparison of NAb responses. NAb data from WT versus mA3 KO mice from Fig. 3 and IFNAR KO versus IFNAR/mA3 dKO mice from Fig. 4c were analyzed. Inoculum doses were lower in the IFNAR KO background to account for the higher susceptibility of these mouse strains to FV infection. Pairwise analyses were performed using a 2-tailed Student’s t test. *p < 0.05; **p < 0.01; NS not significant. [file 12977_2017_349_MOESM1_ESM.docx]

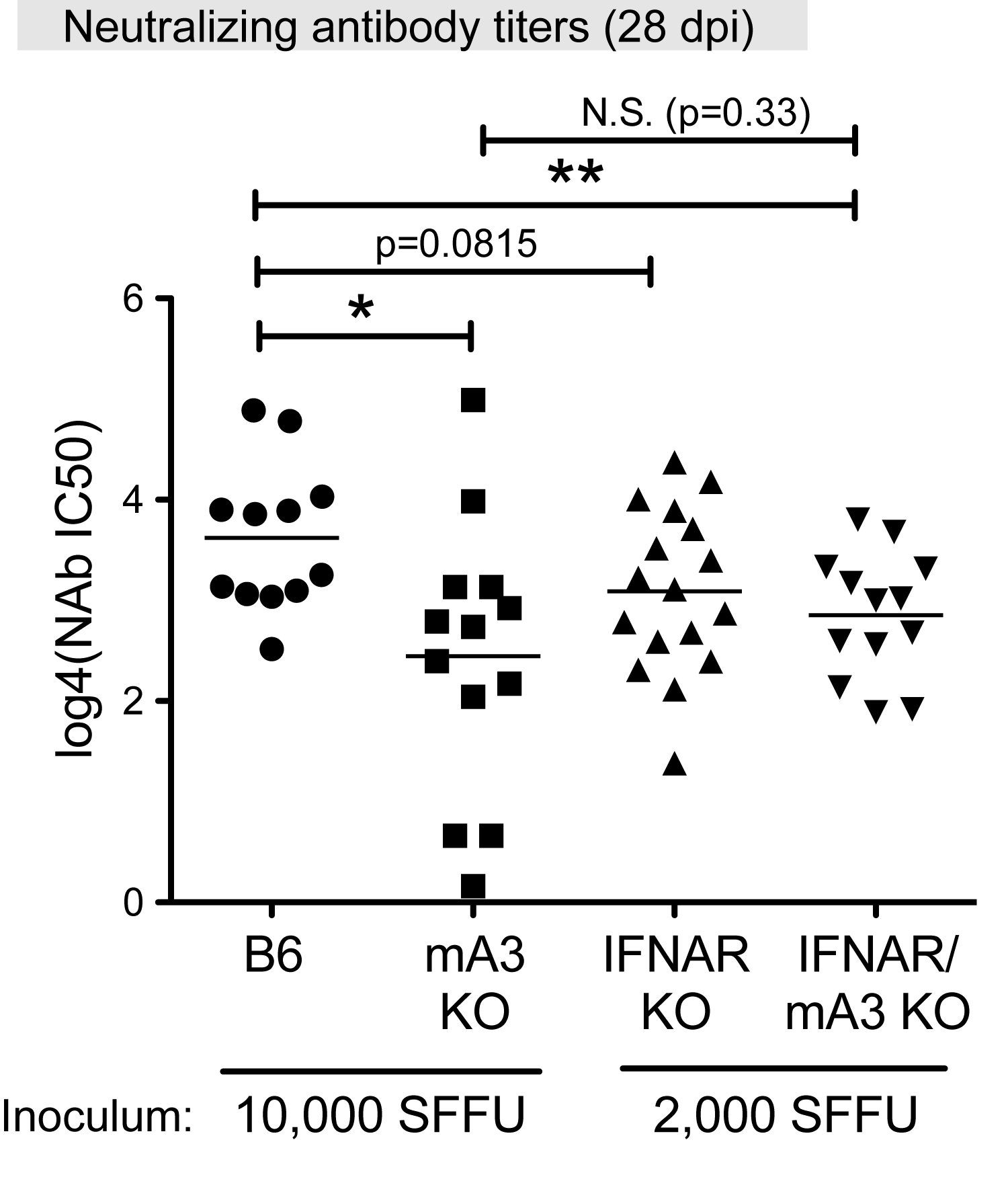


**Supplementary Figure 1.** Four-way comparison of NAb responses. NAb data from WT vs mA3 KO mice from Figure 3 and IFNAR KO vs IFNAR/mA3 dKO mice from Figure 4C were analyzed. Inoculum doses were lower in the IFNAR KO background to account for the higher susceptibility of these mouse strains to FV infection. Pairwise analyses were performed using a 2-tailed Student’s t-test. *, p<0.05; **, p<0.01; NS, not significant.
